# Correction of a Depth-Dependent Lateral Distortion in 3D Super-Resolution Imaging

**DOI:** 10.1371/journal.pone.0142949

**Published:** 2015-11-23

**Authors:** Lina Carlini, Seamus J. Holden, Kyle M. Douglass, Suliana Manley

**Affiliations:** 1 Institute of the Physics of Biological Systems, École Polytechnique Fédérale de Lausanne, Lausanne, Switzerland; 2 Centre of Bacterial Cell Biology, Institute for Cell and Molecular Biosciences, Medical School, Newcastle University, Newcastle upon Tyne, United Kingdom; Universidad Carlos III de Madrid; Instituto de Investigación Sanitaria Gregorio Marañon, SPAIN

## Abstract

Three-dimensional (3D) localization-based super-resolution microscopy (SR) requires correction of aberrations to accurately represent 3D structure. Here we show how a depth-dependent lateral shift in the apparent position of a fluorescent point source, which we term `wobble`, results in warped 3D SR images and provide a software tool to correct this distortion. This system-specific, lateral shift is typically > 80 nm across an axial range of ~ 1 μm. A theoretical analysis based on phase retrieval data from our microscope suggests that the wobble is caused by non-rotationally symmetric phase and amplitude aberrations in the microscope’s pupil function. We then apply our correction to the bacterial cytoskeletal protein FtsZ in live bacteria and demonstrate that the corrected data more accurately represent the true shape of this vertically-oriented ring-like structure. We also include this correction method in a registration procedure for dual-color, 3D SR data and show that it improves target registration error (TRE) at the axial limits over an imaging depth of 1 μm, yielding TRE values of < 20 nm. This work highlights the importance of correcting aberrations in 3D SR to achieve high fidelity between the measurements and the sample.

## Introduction

Optical aberrations compromise the performance of fluorescence microscopes, which can ultimately degrade image quality. Aberrations become more serious in point-localization super-resolution imaging (SR), that is Stochastic Optical Reconstruction Microscopy (STORM)[1] and Photoactivated Localization Microscopy (PALM)[2], where even nanometer-scale distortions can significantly degrade the accuracy of measurements. In SR, a structure of interest is labeled with fluorescent molecules which are imaged and localized with precisions on the order of 10–50 nm in directions lateral to the optical axis and precisions that are typically 3–4 times worse axially [3]. The individual localizations are combined to create a super-resolved image of the structure. To minimize the deleterious effects of aberrations in SR, the localizations themselves require correction.

SR techniques were first extended to three-dimensions (3D) by introducing astigmatism[4] into the imaging system with a cylindrical lens, effectively encoding molecules’ axial positions onto the microscope’s point spread function (PSF) [5]. Astigmatic 3D SR requires correction of unwanted aberrations to faithfully reproduce the dimensions of the target object. To address this, aberrations producing shifts of the perceived location of single molecules along the axial direction and their correction have been described [5–7]. Distortions along the lateral direction have also been reported; specifically, a lateral shift in the centroid of a fluorescent bead’s image depending on its axial position was noted for astigmatic [5], double-helix point spread function (DH-PSF) [8–11] and biplane-based 3D SR imaging. Correction of this error has been previously performed [5, 8–10] by measuring the lateral translation of a bead along the axial direction and subsequently subtracting this shift from localizations. Another work has addressed the issue by using a phase-retrieved pupil function that includes system-specific aberrations at the cost of algorithmic complexity and relatively long fitting routines [12]. Despite these works, this lateral shift has not been systematically characterized and no software tool to correct it has been published. If left uncorrected, such an axial-dependent lateral shift can also impair multicolor registration accuracy over the imaging depth.

In this work, we investigate this optical distortion, which creates an axial dependence on the molecule’s perceived lateral location and explain how it deforms 3D SR data. We find that this distortion is present on four set-ups with different manufacturer lenses and show that it is microscope-dependent; typically, we observe lateral shifts > 80 nm over a ~ 1 μm axial range. We also provide experimental evidence to show that wobble is inherent to many varieties of 3D microscopy. We investigate the source of wobble using phase-retrieval methods and by measuring the distortion under different experimental conditions. Our data suggest that a non-rotationally symmetric optical aberration is responsible for this axial-dependent, lateral shift. We verify that its correction improves the accuracy of 3D localizations to better reflect the true structure and provide an algorithm and software to eliminate this shift. Our software tool only requires an axial stack of localized beads in order to correct wobble, making this method easy to implement. Finally, we demonstrate that the correction tool we provide, combined with a 3D polynomial transformation can deliver a computationally efficient way to register multicolor 3D SR data sets with a < 20 nm target registration error over a ~ 1 μm range.

## Materials and Methods

### Bead sample preparation

Multi-emitting, 100 nm fluorescent beads (excitation/emission: 360/430 nm, 505/515 nm, 560/580 nm, 660/680 nm, TetraSpeck™, Invitrogen) were diluted in ethanol (1:200) and spread onto a coverslip (Menzel-Glaser). Once dried, 13 μm x 13 μm regions containing 5–10 beads were imaged. When the density of single beads was sparse, beads were scanned laterally to sample the full field of view. Imaging was performed in PBS. For long-term use of bead samples, beads were spread onto a 25 mm coverslip with a silicon gasket placed on top. Inside the gasket, a drop of water was placed and the gasket was sealed with an 18 mm glass coverslip.

### *Caulobacter crescentus* sample preparation

A merodiploid strain of CB15N containing both the native *ftsZ* gene and a second chromosomal copy of *ftsZ-dendra2* under the control of the native xylX promoter [13] was grown overnight in liquid M2G media to mid-exponential phase. FtsZ-Dendra2 expression was induced with 0.003% Xylose for 3 hours, and the sample was sandwiched between a glass coverslip and a 2.5% agarose pad as described previously [14]. Samples were then imaged as described below. Trapping the bacteria between the glass surface and stiff hydrogel (high-percentage agarose pad) ensures the bacteria lie flat on the glass. We verified that bacteria lay flat on the glass by inspection of phase contrast images of the cells; tilted cells were not observed.

### Optical setups and 3D localization

Bead imaging to demonstrate the wobble distortion, for dual-color registration experiments and experiments to identify the source of wobble were performed on a modified Olympus IX71 inverted microscope equipped with an Olympus NA 1.40 oil immersion objective. A 641 nm laser (Coherent, CUBE 640) or 561 nm laser (Coherent Sapphire) were directed onto a 4-color dichroic (89100bs, Chroma) and through the objective to excite the bead sample. Fluorescence was collected through the same objective and directed onto an EMCCD camera (iXon+, Andor) with a resulting pixel size of 100 nm. Red (580 nm) or far red (680 nm) light reaching the EMCCD passed through an ET605/52 nm (Chroma) or ET700/75 nm (Chroma) emission filter respectively. An f = 1000 mm cylindrical lens (Thorlabs LK1002RM-A) was used to introduce a slight astigmatism onto the PSF. Z-(axial-)stacks of beads were acquired over a 1 μm range, with a 20 nm z-spacing between subsequent images. The Z position was controlled using a piezo objective scanner (P-725 PIFOC, Physik Instrumente). Note that in experiments where the cylindrical lens was removed, the Z values along the X-axis are axial distances relative to the defined in-focus position (Z = 0), where the PSF is smallest and the most circular. 3D localization was performed with rapidSTORM 3.3 [15], which required a 3D calibration curve to define the PSF x and y width as a function of z. Local Signal-to-Noise-Ratio (SNR) detection with an SNR threshold of 50 was used. Z values were corrected for refractive index mismatch by rescaling by 0.72 [6].

Live cell PALM images of *C*. *crescentus* FtsZ and coverslip tilt experiments were performed on a custom-built microscope equipped with a Nikon NA 1.49 oil immersion objective lens. The objective was mounted to a solid block of aluminum and attached to the microscope stage (ASI RAMM), rather than a moveable turret. This minimized mechanical drift. Sample z- and xy-positions were controlled using a z-piezo stage (ASI PZ-2500) and an automated servo motor stage (ASI). More details on the microscope hardware were described previously [14]. Fluorescence was detected using a 128 x 128 pixel EMCCD camera (Photometrics Evolve 128). Fluorescence was excited at 560 nm (MPB VFL-P-300-560). A 405 nm laser (Coherent, Obis) was used for photoactivation. Cells were imaged with an exposure time of 10 ms. Light reaching the EMCCD passed through a dichroic mirror (ZT561rdc) and an emission filter (Chroma ET605/52M). As before, an f = 1000 mm cylindrical lens (Thorlabs LK1002RM-A) was used to introduce astigmatism.

### Phase-retrieval procedure

The scalar diffraction-based phase retrieval method outlined in references [12, 16] was employed to estimate the microscope pupil function. An image stack of a fluorescent bead deposited on a glass coverslip was captured in the far red channel with a cylindrical lens (Thorlabs LK1002RM-A) in place, over a range of axial positions at 20 nm intervals from -680 nm to + 760 nm. The images were cropped to 41 pixels x 41 pixels and then the background was determined by computing the average of four regions of the in-focus image where there was no signal. This background was subtracted from all images in the stack; pixels with resulting intensities less than zero were reset to zero. The images were then resized by zero-padding symmetrically about the center of the in-focus PSF to 513 pixels x 513 pixels; this allowed us achieve better resolution in the spatial frequency domain.

The phase-retrieved pupil function was obtained by starting from a uniform phase and unit amplitude pupil plane estimate and propagating the field to several points near the image plane using a fast Fourier transform and the defocus kernel used in [16]. The microscope was modeled as having a numerical aperture of 1.4, a pixel size of 100 nm and immersion oil with refractive index of 1.51. The wavelength was assumed monochromatic with a wavelength of 670 nm; the monochromatic assumption did not appear to adversely affect the agreement between the experimental PSF and that derived from the phase-retrieved pupil function. For each iteration of the procedure, the spatially-dependent amplitudes of the propagated field at planes located at -680 nm, -250 nm, 0, +250 nm, and +680 nm about the image plane were replaced by the square root of the signal in the corresponding processed image stacks. These fields were then inverse Fourier transformed and divided by the defocus kernel to obtain five new pupil function estimates. The five separate pupil functions were averaged to produce the resulting pupil function estimate for that iteration of the phase-retrieval loop. 30 iterations were used in total, though good convergence was observed before this point.

The phase-retrieved PSF stacks were then computed by propagating the phase-retrieved pupil to axial planes between -680 nm and +760 nm in 20 nm steps. Zernike polynomial fitting to both the amplitude and phase of the phase-retrieved pupil was performed using the third party ZernikeCalc function for MATLAB[17].

### Wobble calibration and correction algorithm

The observed lateral shift in bead position as a function of *z* was measured for a sample of 0.1 μm fluorescent beads (TetraSpeck™, Invitrogen), excited at 560 nm. A Z-stack over a 3 μm range was acquired in 20 nm steps. Observed bead position was measured using the rapidSTORM software. The observed lateral displacement of bead localization as a function of *z* was fitted to a cubic B-spline. The best fit splines for each bead were then combined using a second spline fit, resulting in the final calibration curve. Bead aggregates do not accurately encode axial information and thus, give rise to outlier spline fits. These are excluded from the final calibration curve.

For subsequent datasets, the resulting best-fit lateral displacement (‘wobble’) curve was subtracted from each observed localization *xy* position based on observed *z* position. MATLAB scripts to carry out wobble calibration and correction are provided (S1 File *)*.

### Dual-color, 3D SR registration

The modified Olympus IX71 microscope described in *Methods* contains a commercially available image splitter (Cairn Optosplit). With a dichroic inserted (89100bs, Chroma), red and far red fluorescence light is directed to the EMCCD, where it is physically separated. A snapshot of a nanogrid (Miraloma Tech.) is acquired and cropped to form a red and far red grid image. This grid is a coverslip made up of an array of holes, 200 nm in diameter and spaced 2 μm apart. Using a custom-written MATLAB program (2013a) the red image was mapped onto the far red image. Briefly, grid images are localized in 2D and then coarsely aligned. Each localized image contains points corresponding to the positions of grid holes; matching hole localizations from the two channels make up control pair points. By assessing nearest neighbor distances, mismatched control points are discarded. Finally, coarsely aligned control pair points are used to define an LWM transform using a built-in MATLAB function (cp2tform, ‘LWM’). The initial and LWM transforms are then applied to the wobble corrected data from the red channel. An axial displacement to account for the z chromatic shift is applied to the red channel when data is localized in 3D; this is a direct consequence of having a z-calibration curve in the red channel shifted from that of the far red. To account for small shifts introduced by changing the sample, we applied a final translation. A pair of control pair points was selected to calculate a linear shift in x and y, which was later applied to beads in the red channel.

Once pairs of images were wobble corrected and registered, the target registration error (TRE) is determined by calculating the total distance (both axial and lateral) between control pair points. Beads that were used to define the z-calibration and wobble-calibrations are not used when calculating TRE values.

## Results and Discussion

### Quantifying and correcting wobble

The optical distortion we describe introduces a depth dependence on the molecule’s observed lateral location, which we will subsequently refer to as ‘wobble’. Specifically, a molecule above or below the focal plane appears laterally displaced (Fig 1A right) from its true position (Fig 1A, left), with the magnitude of this displacement depending on its depth. This distortion is problematic since it results in axial warping of fluorescently labeled biological structures (Fig 1A). We quantified this effect on our 3D-astigmatic imaging system by acquiring z-stacks of 100 nm fluorescent beads distributed over the field of view. An f = 1000 mm cylindrical lens was placed close to the Fourier plane of a 4f system attached to the microscope camera port as previously described[5]. Once beads from each image of the z-stack were localized in 3D (Materials and Methods), we calculated the lateral displacement of their positions relative to beads in the focal plane; this data was then spline fit to yield wobble calibration curves. In particular, these calibration curves show an average displacement in the x- and y-directions as a function of z; measurements from several beads dispersed over the field of view were taken to yield an average value (Fig 1C, green and magenta solid lines). Since curves from single beads do not differ significantly (Fig 1C, solid versus dotted lines), we reason that the wobble is not field dependent, at least when imaging the 13 μm x 13 μm area centered about the optical axis. Strikingly, the magnitude of this distortion on our system is on the order of 100 nm in a single direction over a 1 μm range in z (Fig 1C). Even at z-ranges of ~ 125 nm, the magnitude of the lateral shift is ~ 20 nm in a single direction. This highlights the need to correct 3D SR data of even thin samples as this is roughly the localization precision of single molecules. Once the system’s wobble is calibrated, we subtract the measured offset from subsequent localizations. Wobble curves before and after applying this correction on fluorescent beads demonstrate its effectiveness (Fig 1C and 1D). The corrected lines show little deviation from the lateral position at the focal plane over a range of 1 μm in z. The accuracy of this correction, estimated from the error bars is ≤ 6 nm.

**Fig 1:**
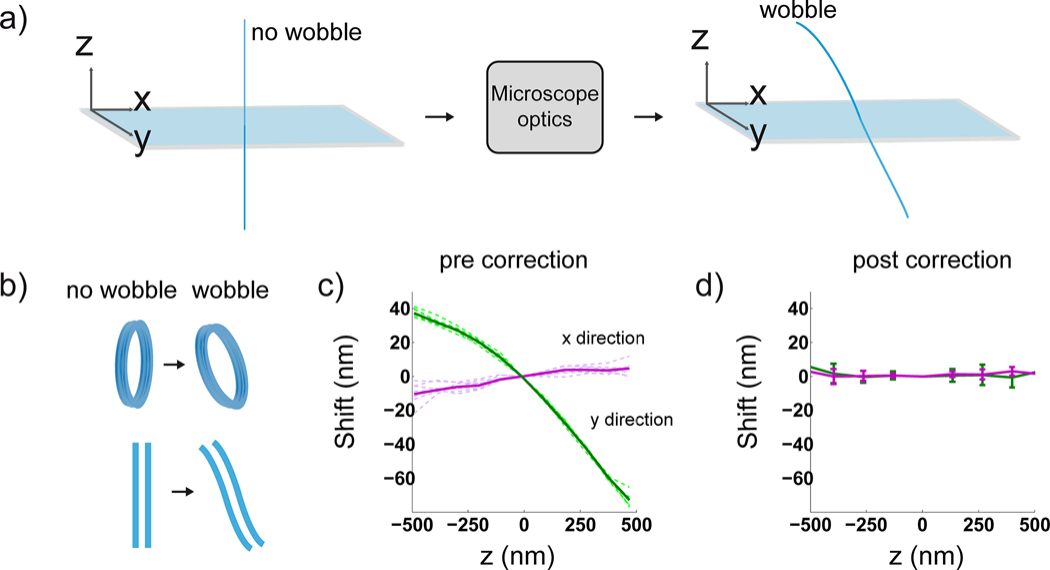
Observed Z-dependent lateral PSF shift (wobble). (a) A wobble-free system is denoted by the straight, light blue line (left) as compared to a system with wobble (right). The wobble effect shown in a) causes a lateral displacement of localizations as a function of axial displacement due to unwanted aberrations introduced by the microscope system. (b) Cartoon of true structures (left) and resultant warped structures (right) imaged on a system with wobble. (c) The lateral shift (or wobble), in the x and y directions as a function of the axial position (z). Dashed, green and violet lines represent data from 10 different beads over the field of view. The green and magenta solid lines represent the average value based on all 10 beads in the y and x-directions respectively. We observe a more significant shift in the y-direction, which is ~ 10 times greater than that in the x-direction. (d) Wobble curves after our correction method is applied; accuracy of correction according to error bars ≤ 6 nm. Error bars are standard deviation (S.D) values of the shift. Maximum S.D for curves in 1c: 6 nm. All data was collected on a modified Olympus IX71 inverted microscope equipped with an Olympus NA 1.40 oil immersion objective.

We tested the robustness of the wobble correction by comparing calibrations from three different days, with three different bead samples, on a single microscope (Fig 2). On the third day of measurement, the cylindrical lens was removed and put back into place; no major adjustments were made to the setup. Repeated measurements on separate days did not produce significant variation in the magnitude and direction of the wobble. Removal and reinsertion of the cylindrical lens in the microscope produces a small change in observed calibration which was within error of the previous days’ measurements. The overall direction remained unchanged. These observations suggest that minor changes to the microscope system, such as changing samples, result in little-to-no change to the wobble calibration. On the other hand, major changes to the system, such as a re-alignment or repositioning of an optical element, will likely require another calibration. Next, to test whether this distortion is exclusive to a single microscope, we took the same measurement on separate systems and found a similar effect (Fig 3A and 3B). One of these systems was a commercial biplane microscope (Fig 3A); thus, demonstrating that wobble is not exclusive to astigmatic 3D systems. Similarly, we observed the distortion in a widefield axial image stack of fluorescent beads (Fig 4A, green curve) from a different widefield fluorescence microscope. In principle, any imaging modality relying on the widefield imaging of single molecules may be affected and should be corrected for wobble, including astigmatic, DH-PSF, and biplane 3D SR. Overall, we noticed this distortion on four different systems equipped with objectives from some of the major manufacturers: an NA 1.49, 100X Nikon, an NA 1.40, 100X Olympus, an NA 1.42, 60X Olympus and an NA 1.46, 100X Zeiss. Three of these microscopes were of commercial origin and one was custom-built.

**Fig 2:**
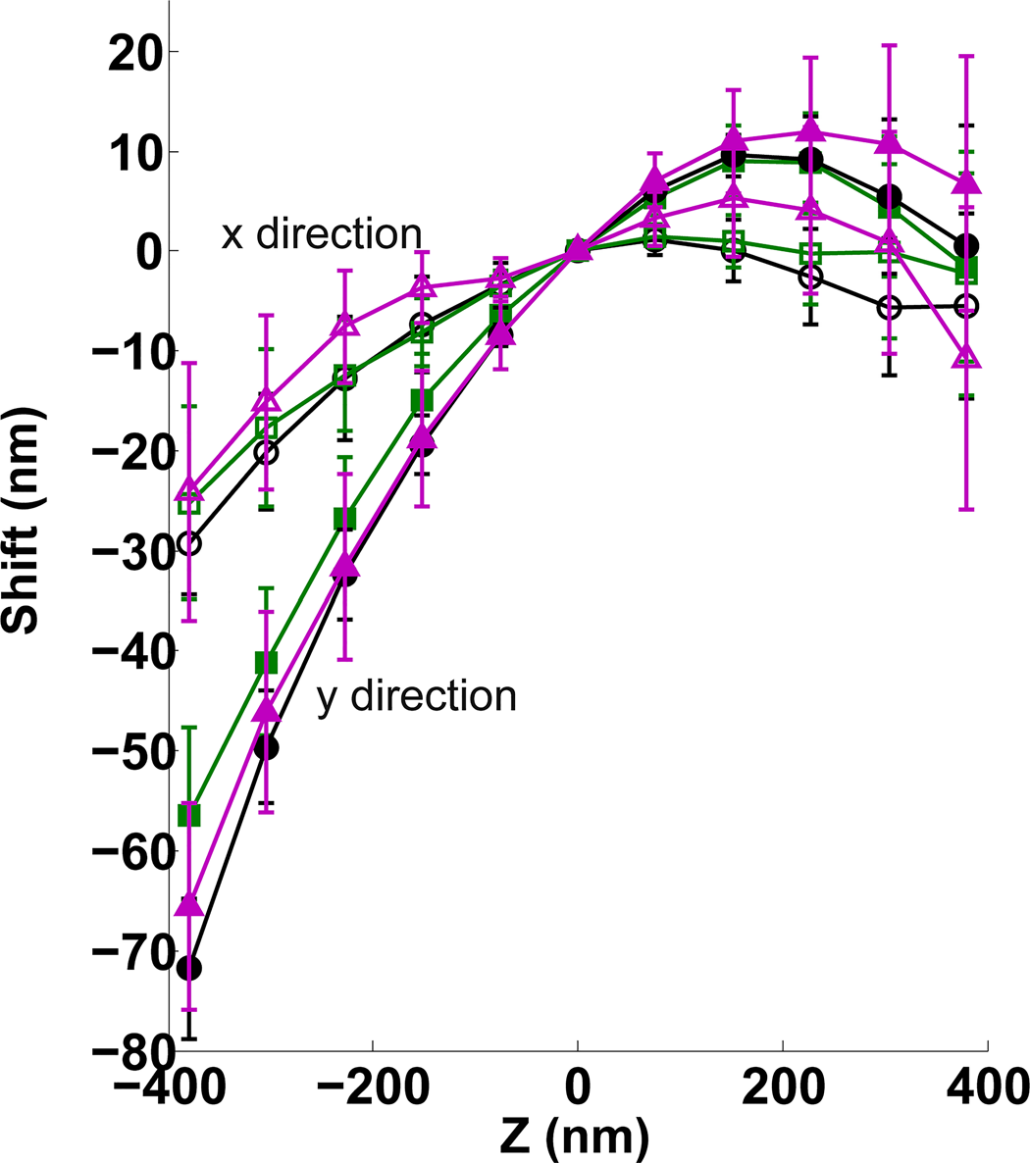
Wobble calibration measured on three days on the same system. Day 1 (black, x-direction: open circle, y-direction: filled circle) and day 2 (green, x-direction: open square, y-direction: filled square) show little deviation from one another. Before measurement on day 3 (magenta, x-direction: open triangle, y-direction: filled triangle), the system’s cylindrical lens was removed and replaced.

**Fig 3:**
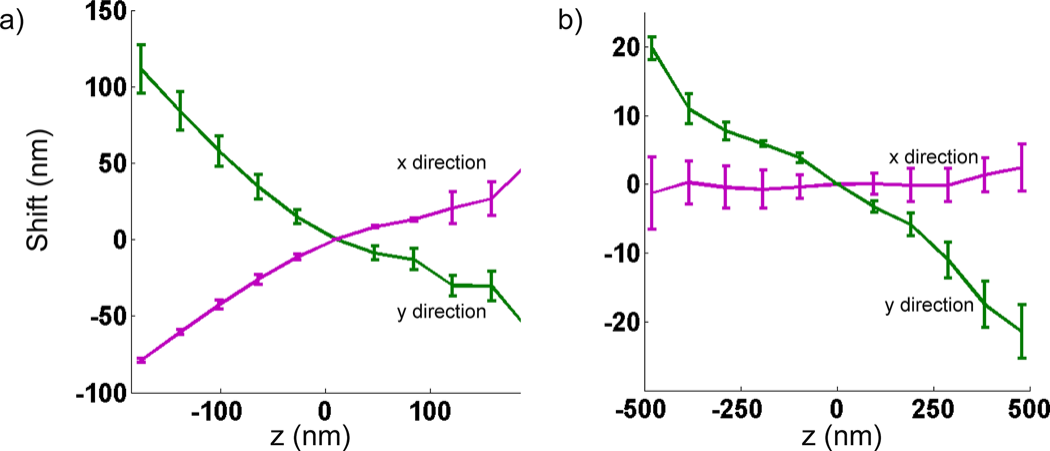
Wobble effect on two separate systems. (a) Wobble demonstrated on a commercial biplane setup equipped with an NA 1.42, 60X water immersion objective (UIS2 Plan Apochromat Olympus). **(**b) Wobble demonstrated on a system, equipped with a cylindrical lens for astigmatic, 3D imaging and a Zeiss 100X objective (Plan-Apo NA 1.46). Measurement from 4–5 beads. Error bars are standard deviation (S.D) values of the shift value.

**Fig 4:**
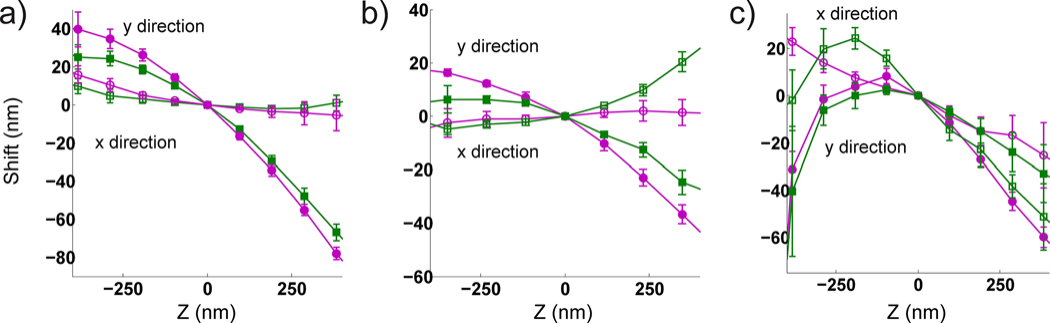
Wobble distortion under different experimental conditions. **(**a) Wobble curves in the presence (magenta) and absence (green) of a cylindrical lens, showing little difference in magnitude and direction. (b) Wobble curves at two different objective rotations 45° apart (0°, magenta, 45°, green), showing changes in both directions and magnitude. (c) Wobble curves with 0° (magenta) and ~ 1.5° (green) coverslip tilt. Measurements from 5–6 beads. Error bars are standard deviations of the shift value. X-directions: magenta, open circle, green, open square and y-directions: magenta, filled circle, green, filled square in unmodified (with cylindrical lens, no coverslip tilt and no objective rotation) and modified positions respectively.

### Investigating the source of wobble

Previous studies [11, 18–20] have shown that the dipole orientation of single molecules leads qualitatively to the same centroid shifts that we describe. However, this is an unlikely explanation for the source of the wobble effect because it is observed in images of sub-diffraction fluorescent beads, which may be modeled as isotropic emitters composed of many randomly oriented dipoles [19]. Others have demonstrated that a discrepancy between the 2D Gaussian PSF of the fitting model and the actual PSF, which includes aberrations, can lead to inaccurate fitting[21]. Indeed, a mismatch between the 3D PSF fitting model and the actual aberrated PSF could lead to the axially dependent lateral shift we observe. We explored possible sources of the aberration(s) associated with the wobble on our modified Olympus IX71 microscope. By rotating the objective lens about its fixed axis, we found that the direction of the wobble changes (Fig 4B). Importantly, since rotation does not affect tilt of the objective, rotation about the objective’s axis would not introduce a change in the wobble’s direction if the effect were solely due to an objective or another optic that was tilted with respect to the optical axis. We also tested whether the presence of the cylindrical lens affects the wobble and found that it has little impact on both its magnitude and direction (Fig 4A). Finally, we tested whether coverslip tilt affects wobble. We induced an ~ 1.5° coverslip tilt by adjusting the grub screw height on one side of the microscope stage; this is similar to the angle in reference [22], where coverslip tilt led to a wobble-like distortion. We compared wobble curves with and without coverslip tilt and found significant differences in magnitude and direction (Fig 4C). Although coverslip tilt contributes to the wobble, it is not the sole cause since the distortion remains without coverslip tilt (Fig 4C, magenta curves with open and closed circles). Our data thus suggest that coverslip tilt and a non-rotationally symmetric optical aberration inherent to the objective lens contribute to the wobble. Conveniently, if a small amount of coverslip tilt and wobble were both present in a system, our computational method will simultaneously correct both.

We computationally verified this hypothesis by estimating the pupil function of our modified Olympus IX71 microscope using scalar diffraction theory and a previously described phase retrieval procedure (Methods). The phase and amplitude of the phase-retrieved pupil function were separately fitted to the first 91 Zernike polynomial terms ordered by their Noll indices[23]. We set the coefficients of the non-rotationally symmetric terms to zero and recomputed the PSF stack (Fig 5). The non-rotationally symmetric components have azimuthal terms that vary as the sine or cosine of the azimuthal pupil coordinate. This step was further motivated by the observation that aberrations such as astigmatism, which do not display axial shifts with defocus, are described by terms possessing rotational symmetry; those that do have axial shifts, such as coma and its higher orders [24], lack rotational symmetry. Wavefront tilt is the only exception. It is not rotationally symmetric, but it does not impart any distortion onto the PSF in either the axial or lateral directions. Removal of only these Zernike polynomial terms from the amplitude and phase of the pupil function results in a wobble-free axial PSF (Fig 5, bottom left). These results suggest that, within the context of shift-invariant scalar diffraction theory, the presence of coma-like aberrations in the pupil plane will lead to an axial dependence of the image’s peak position, and therefore a violation of the assumption used in astigmatic imaging.

**Fig 5:**
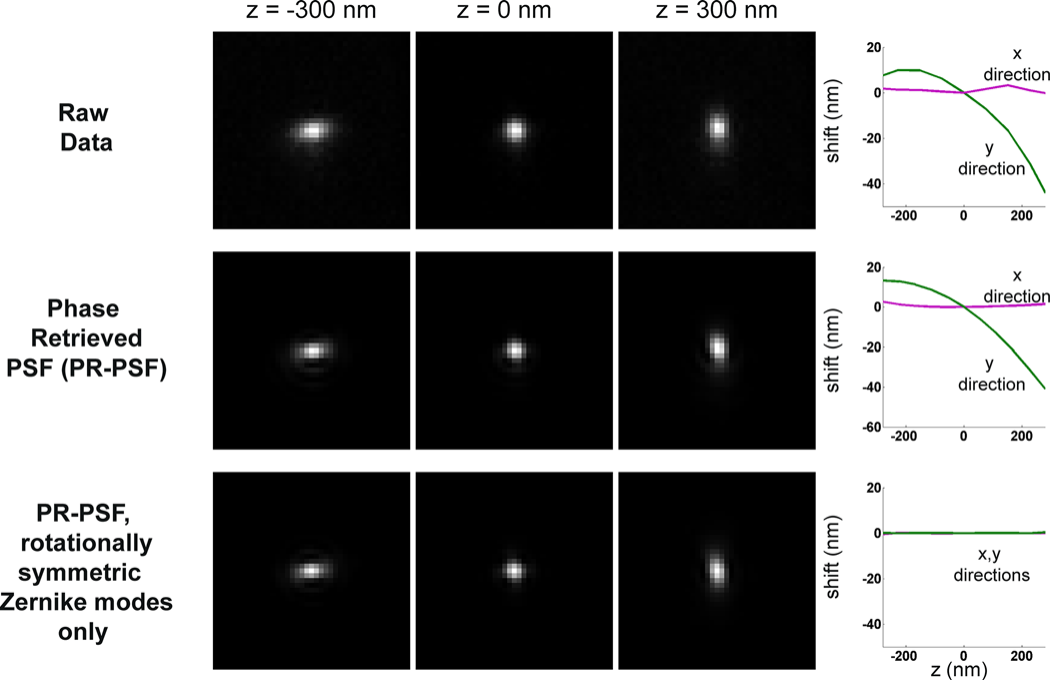
Aberrations that lack rotational symmetry produce the wobble effect. (Top row) Raw data shown at three different axial depths and corresponding wobble curves. (Middle) Phase-retrieved PSF (PR-PSF) at three axial depths and corresponding wobble curves, which are in good agreement with the raw data. (Bottom) PR-PSF with only rotationally symmetric Zernike modes. Resulting wobble calibration shows an absence of the wobble distortion.

At this point it is important to note that any real microscope will possess multiple types of aberrations to varying degrees, which can include non-rotationally symmetric aberrations due to the conditions in the object space such as coverslip tilt. For these reasons, the magnitude and nature of the wobble varies with the microscope and imaging conditions as well. Furthermore, we do not expect the assumption of the position-invariance of the wobble to hold when imaging larger fields of view because the position-invariance of the PSF is an invalid assumption in large-field imaging systems[25].

### Application to FtsZ ring-like protein in bacteria

To demonstrate the experimental utility of the wobble correction, we acquired 3D SR images of the bacterial cytoskeletal protein FtsZ in *C*. *crescentus*, which is observed by cryo-electron microscopy [14, 26] to form a vertically oriented ring-like structure at mid-cell during division (Fig 6). Without wobble correction, 3D SR images of Z-rings show significant vertical tilt along the Z-axis (Fig 6B). Once a wobble correction is applied (Fig 6C), the FtsZ ring becomes parallel to the axial direction and thus more accurately reflects the underlying structure. The calibration curve used for correction of this sample was generated using a fluorescent bead stack. Since wobble is eliminated from biological data (Fig 6) using a calibration measured from fluorescent beads, we can conclude that this distortion is primarily system-, rather than sample-induced.

**Fig 6:**
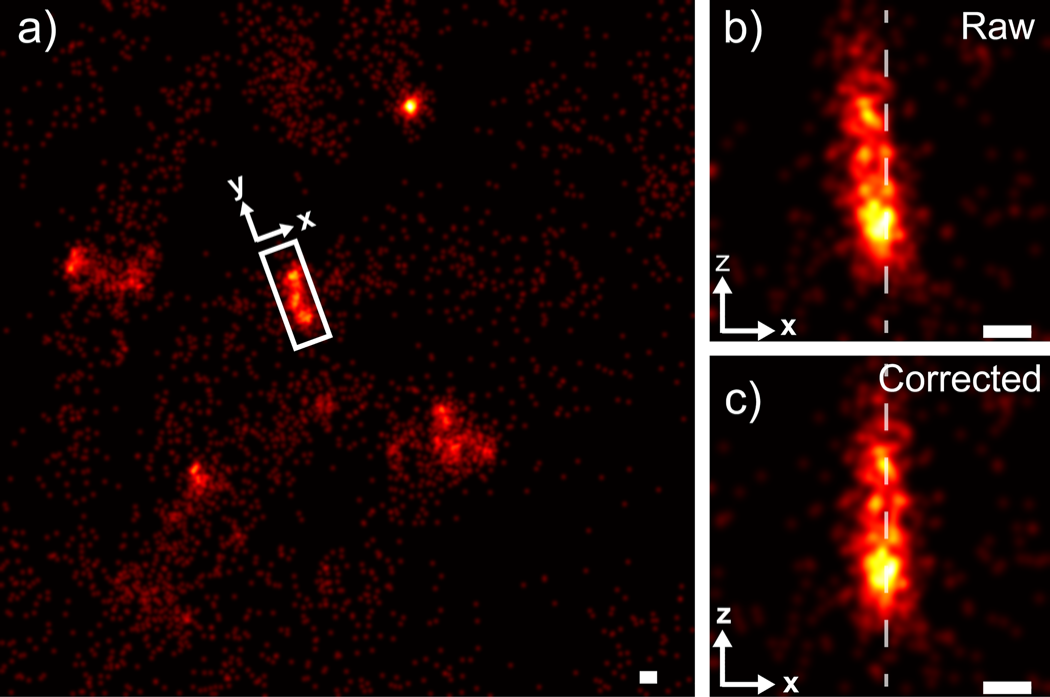
Wobble correction applied to an exemplar FtsZ ring in *Caulobacter crescentus*. (a) A field of view containing several bacteria (b) Zoom of the boxed region in a, containing raw localizations of the FtsZ ring, having a tilt of ~ 9.0° (c) The same FtsZ ring as in b after application of the wobble correction with a < 1° tilt. Dotted lines in b and c guide the eye to visualize the tilt of the FtsZ ring. Scale bars: 100 nm. All data, including wobble calibration, was collected on a custom-built microscope equipped with a Nikon NA 1.49 oil immersion objective lens.

### Application to dual-color, 3D registration

We developed a procedure to accurately register 2-color, 3D SR data, which incorporates our wobble correction. When combining multicolor SR datasets, the registration error should be minimized to preserve the nanometric accuracy of these measurements. However, with wobble present in each channel, (Fig 7A) the registration error significantly worsens as a function of z (Fig 7B). This gives rise to an axial dependence of the colocalization accuracy, which depends on how much the magnitude and direction of the wobble differ between channels. To overcome this problem, we correct the wobble in both channels before registration using a 2D local weighted mean (LWM) transformation. (Fig 8, Materials and Methods).

**Fig 7:**
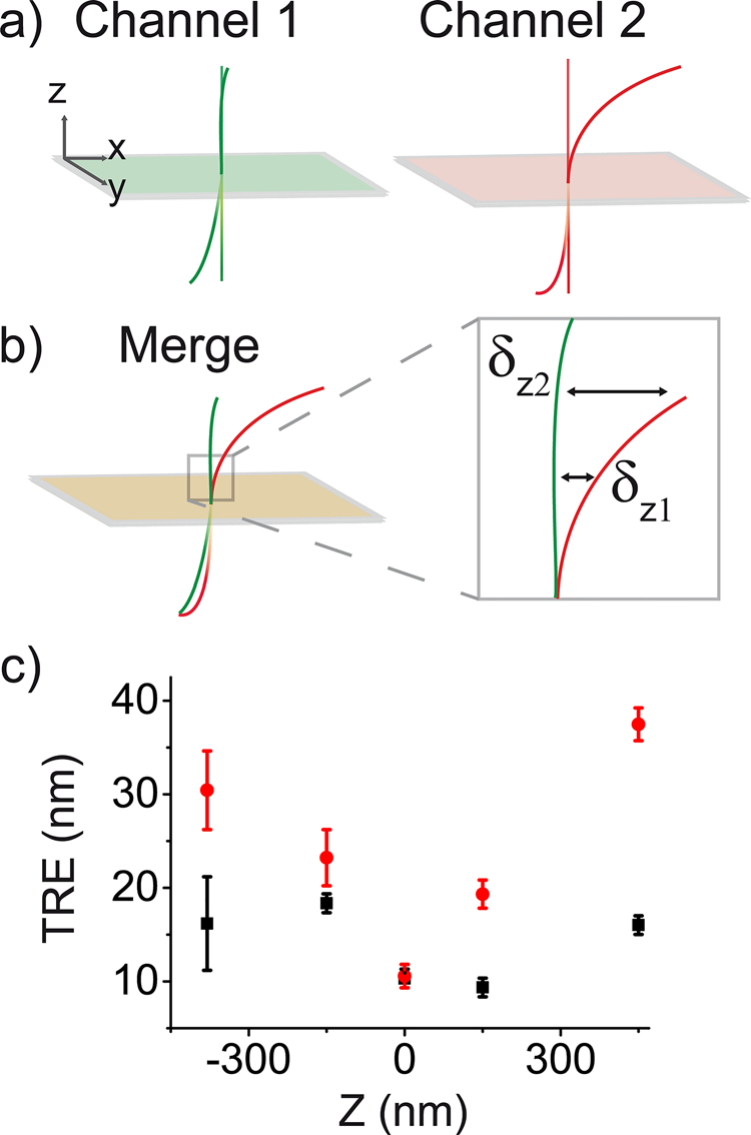
Application of wobble correction for 2-color, 3D SR image registration. (a) A cartoon of the wobble effect in 2 independent channels is shown. Green and pink rectangles represent focal planes. Straight lines reference the wobble-free system. (b) Effect of merging channels: since the wobble differs over z, this increases the registration error *δ_z_* where wobble curves differ most, which is typically at the z-limits of the imaging depth (c) the target registration error (TRE) as a function of z for a locally weighted mean (LWM) transformation defined at the focal plane (red data) and a LWM transformation defined at the focal plane with wobble correction included (black data). Error bars are standard deviation of TRE values. Measurements taken from 4 beads over the field of view.

**Fig 8:**
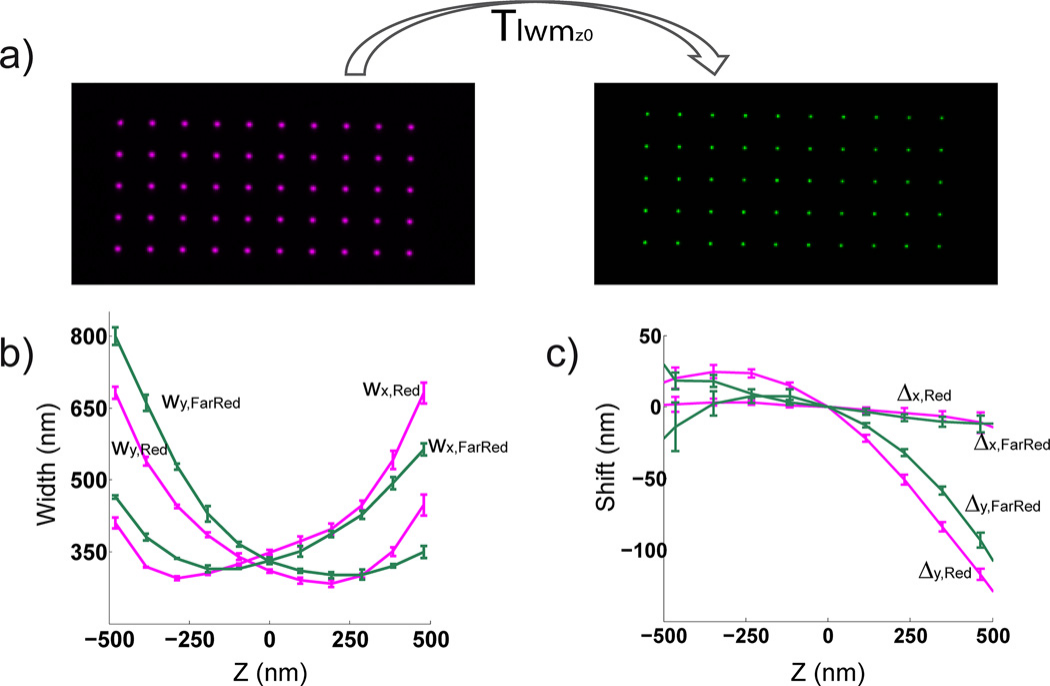
Workflow of dual color, 3D registration. Work flow for image registration of 3D SR data. (a) At the focal plane (z0), a local weighted mean transformation T_lwmz0_ is defined using a nanogrid. This allows us to map one color channel onto the other. (b) After data is acquired, it is fit to a 3D calibration curve from its channel to obtain z information and to correct the chromatic shift in z (magenta, red channel z calibration; green, far red channel z calibration), then (c) 3D data from each channel (magenta, red channel; green, far red channel) is wobble corrected. Once both the z and wobble calibrations are applied to the data from each channel, T_lwmz0_ is used to map one channel onto the other. Measurements from 10 beads shown in b and c.

To assess the error obtained with our registration procedure, we quantified the target registration error (TRE) as a function of z using 100 nm fluorescent beads. Bead images from a red (600/75 nm emission) and far red (700/75 nm emission) channel were registered (Fig 8). We computed the magnitude of the wobble correction by plotting the TRE versus z for data subject to a 2D LWM transformation and data that was additionally wobble corrected (Fig 7C). We see improved TRE values at the z-limits, where the wobble curves differ most (Fig 8). At the z limits, the TRE decreases by a factor of ~ 2–3 in the case where wobble correction is implemented in the registration procedure. We also noticed that the TRE values were < 20 nm over ~ 1 μm axially. These average TRE values are on the order of typical lateral localization precisions, so we expect this registration procedure to be sufficient for most applications.

Recently, a *3D* LWM algorithm was shown to accurately overlay 3D SR data with < 10 nm precision [10]. When localization precisions of less than 10 nm are not required, our registration method is computationally more efficient since it uses a 2D instead of a 3D LWM transformation. Acquiring data to define the transformation for our method is also faster since it requires a snapshot rather than a scan throughout the entire 3D field of view.

## Conclusion

We demonstrate that correcting “wobble” in astigmatic 3D SR microscopy reduces distortion both for test and biological samples. We provide an algorithm and software to carry out this correction. Further, we show that ‘wobble’ is a prevalent aberration, since it was present in multiple microscopes and objectives from different manufacturers that we examined. Since the magnitude and direction of the wobble differs from system to system (Figs 1 and 2), we can conclude that this is a microscope-dependent distortion. Our experiments suggest both coverslip-tilt and a non-rotationally symmetric aberration inherent to the objective can both contribute to the source of wobble. We further explored the source of this distortion by determining our system’s PR-PSF. When aberrations that lack rotational symmetry in the pupil function were removed from the PR-PSF, we eliminated wobble; therefore, aberrations which lack rotational symmetry in the pupil function are responsible for the wobble. Consistent with our observations, recent work[27] highlights the need to eliminate objective-induced aberrations in SR. Adaptive optics has proven to be advantageous in correcting objective and sample-induced aberrations [28, 29], however its implementation can be costly and complex. Our wobble correction software provides an effective method for accurate single and dual color 3D SR microscopy, which is useful for biological applications where accurate 3D structure, rather than just colocalization, is important. This correction tool will also be valuable to 3D correlative microscopies that include SR such as correlative PALM/electron microscopy [30]. Here, accurate 3D shape from the PALM image is required for alignment, since features from this fluorescent image are used for registration [30].

We observed a non-field-dependent wobble; however, when moving to systems with larger fields of view, field-dependent wobble should become significant. This correction can be achieved with our algorithm by introducing a lateral dependence into the correction in addition to the current axial dependence. In the future, we expect that establishing a detailed understanding of the source of the optical aberrations described here will facilitate the design of more accurate super-resolution microscopes.

## Supporting Information

S1 File Supplementary Software for wobble correction. Matlab wobble correction with instructions and example data.
(ZIP)
